# A retrospective study on the effect of the COVID-19 pandemic on dental treatments in adults

**DOI:** 10.1186/s12903-022-02160-y

**Published:** 2022-04-12

**Authors:** Diego Gómez-Costa, Juan Manuel Ramírez, Iván García Guerrero, Giovanni Giovannini, Rosa Rojo, Rafael Gómez-de Diego

**Affiliations:** 1 Doctoral Program in Health Sciences. Faculty of Health Sciences, Rey Juan Carlos University, Avenida de Atenas s s/n, 28922 Alcorcón, Madrid, Spain; 2 Department of Morphological Sciences, School of Medicine, University of Córdoba, Avenida Menéndez Pidal, 7, Córdoba, Spain; 3 Department of Nursing and Stomatology, Faculty of Health Sciences, Rey Juan Carlos University, Avenida de Atenas s/n, 28922 Alcorcón, Madrid, Spain; 4 Faculty of Dentistry, Alfonso X el Sabio University, Villanueva de la Cañada, 28691 Madrid, Spain

**Keywords:** COVID-19, SARS-CoV-2, Oral health, Pandemic, Lockdown, First wave, Dental emergency

## Abstract

**Background:**

The aim was to analyze the prevalence of dental treatments that were not performed in a dental care university referral center in the capital of Spain during the first wave of the COVID-19 pandemic.

**Methods:**

This was a retrospective observational study based on the registry of medical records. Sex, age, nationality, and the type of treatment that was not performed in the service of the Integrated Adult Dental Clinic subject of the Dentistry degree at the Rey Juan Carlos University of Madrid were analyzed.

**Results:**

A total of 392 medical records were analyzed. The prevalence of the treatments that were not performed was 58.67% (95% CI 53.74–63.44) of conservative treatments, 47.45% (95% CI 42.55–52.39) of periodontal treatments, 27.30% (95% CI 23.12–31.91) and 13.52% (95% CI 10.49–17.26) of clinical activities. The patients most affected by the absence of dental treatment ranged in age from 35 to 74 years. Age, sex, and nationality were not influential in not performing dental treatments.

**Conclusions:**

The COVID-19 pandemic could have negatively influenced treatments, such as conservative and periodontal treatments, that increasing the risk of tooth loss in adults.

## Background

Severe acute respiratory syndrome due to coronavirus 2 (SARS-CoV-2) of the Coronaviridae family causes COVID-19 disease [1]. It has a high contagion capacity and has caused a large number of deaths worldwide [2]. Manifestations of the disease range from asymptomatic development of the disease, mild symptoms, or severe pneumonia [3]. In the field of dentistry, the oral manifestations of the disease are cluded ulcer, erosion, bulla, vesicle, pustule, fissured or depapillated tongue, macule, papule, plaque, pigmentation, halitosis, whitish areas, hemorrhagic crust, necrosis, petechiae, swelling, erythema, and spontaneous bleeding [4]. On the other hand, dentists can be the first line of diagnosis of the disease since they work in close contact with patients and are at risk of being affected by COVID-19 and other respiratory infections [5].

The main routes of transmission of COVID-19 are Flügge droplets (size greater than 5 microns) that are emitted when speaking, coughing, or sneezing [6] and Wells droplets (diameter less than 0.1 microns), with less possibility of transmission from microorganisms. Likewise, transmission can be occur through direct contact of the hands with contaminated fomites, the bucconasal or ophthalmic mucosa [7] or feces [8].

The infection that was first reported in the city of Wuhan (People's Republic of China) in December 2019, became a global public, social, and economic health problem [9], and was declared a pandemic by the World Health Organization (WHO) on March 11, 2020, due to the levels of spread and severity of the disease [10]. During the same month, the Ministry of the Presidency of Spain published Royal Decree 463/2020 in the Spain's Official State Gazette (BOE), declaring a state of alarm to manage the health crisis caused by COVID-19 [11]. The Ministry of Health, Consumption and Social Welfare (SCBS) issued order SND/310/2020, on March 31, which established specific health centers, services, and establishments as essential services, whereas dental clinics did not have this consideration [12].

Worldwide, in the first peak of the pandemic, many dental clinics significantly reduced treatments or stopped their activity [13], limiting themselves to nondeferrable emergency care [14]. Dental repercussions have led us to analyze the experiences of dentists [15, 16], evaluate the general approach against the virus [17, 18], and study its clinical, legal, and economic consequences [19]. The full extent of damage due to the suspension of dental care can be assessed in the number and types of treatments that were not performed on the patient.

In the capital of Spain, the Fundación Clínica Universitaria of the Rey Juan Carlos University (FU-URJC) of Madrid is one of the most important academic and health care centers of public reference in the southern area of the city, in terms of the number of patients treated. Due to the regulations described, this center ceased dental care activity, only performing treatments associated with dental emergencies in the first wave of the pandemic.

The aim of this study was to analyze the prevalence of dental treatments that were not performed in a university reference center for dental care in the capital of Spain during the first wave of the COVID-19 pandemic.

## Methods

A retrospective observational study was conducted following the Strengthening the Reporting of Observational Studies in Epidemiology (STROBE) recommendation guidelines [20].

### Study context and participants

The treatment plans included the clinical records registered during the 2020–2021 academic year (from September 4, 2020, to April 30, 2021) for the Integrated Dental Clinic for Adults of the fifth year of the Degree in Dentistry was analyzed from Rey Juan Carlos University (URJC), Madrid (Spain), attended by fifth-year students of the Degree in Dentistry.

Subjects of any sex, nationality, aged ≥ 18 years, and in good general health were included. The excluded participants received other health care services from the same entity related to the Dental Clinic for Special Patients and Gerontology and the Integrated Children's Dental Clinic of the Degree in Dentistry.

### Variables

The participants' data were collected from the electronic medical records of the URJC University Clinic Foundation by two operators in an independent and anonymized manner.

The variables recorded were age (quantitatively and categorically, following the proposed age ranges of the Spanish National Health Survey [21] divided into the following ranges: 18–24 years, 25–34 years, 35–44 years, 45–54 years, 55–64 years, 65–74 years, 75–84 years, 85 years and older), sex (male or female), nationality (Spanish or foreign) and planned treatments that were not carried out due to the COVID-19 pandemic: conservative treatments, prosthetic treatments, periodontal treatments and clinical activity (such as the first visit, check-ups or other activities not included above).

### Sample size

For the sample calculation, the Wald test was performed to compare the proportion of treatments that were not carried out with a hypothetical reference value (90% of the treatments under standard conditions), using a power of 0.8 and a significance value of 0.05, with the estimated effect size of delta = 0.0349.

### Statistical analysis

Descriptive statistics of the quantitative and qualitative variables were carried out. The Shapiro–Wilk test was performed to analyze the normality of the data. For the calculation of the confidence interval of the prevalence, the Wilson method was chosen. Multinomial logistic regression was performed to evaluate the influence of sex, age group, and nationality on the number and type of treatments that were not carried out. All statistical tests were performed with a 95% confidence level using Stata 16.1 software (Stata Corp, College Station, TX, USA).

## Results

Three hundred ninety-two medical records were analyzed, and the participants were 220 women (56.12%) and 172 men (43.88%), with a mean age of 52.62 years (SD = 0.85). Participants of foreign nationality represented 7.14% of the sample (Table 1).

**Table 1:** Descriptive statistics of sex and nationality according to the age range; %: of the total sample

Age range (years)	Sex				Nationality			
	Women	%	Men	%	Spanish	%	Foreign	%
18–24	13	3.32	17	4.34	26	6.63	4	1.02
25–34	19	4.85	14	3.57	28	7.14	5	1.28
35–44	35	8.93	27	6.89	54	13.78	8	2.04
45–54	57	14.54	34	8.67	84	21.43	7	1.79
55–64	35	8.93	25	6.38	58	14.80	2	0.51
% CI65–74	43	10.97	40	10.20	82	58.00	1	0.26
75–84	18	4.59	14	3.57	31	82.00	1	0.26
≥ 85	0	0.00	1	0.26	1	31.00	0	0.00
Total	220	56.12	172	43.88	364	92.86	28	7.14

In order of frequency, the prevalence of the least performed treatments in the entire sample analyzed were conservative treatment (58.67%), periodontal treatment (47.45%), prosthetic treatment (27.30%), and clinical activities (13.52%) (Table 2).

**Table 2:** Prevalence of the types of dental treatment that were not performed

Type of treatment	Treatment not performed (F)	Prevalence (%)	95% CI
Clinical activity	53	13.52	(10.49–17.26)
Conservative	230	58.67	(53.74–63.44)
Prosthetic	107	27.30	(23.12–31.91)
Periodontal	186	47.45	(42.55–52.39)

CI: Confidence interval; F: Frequency; %: Prevalence

**Fig. 1:**
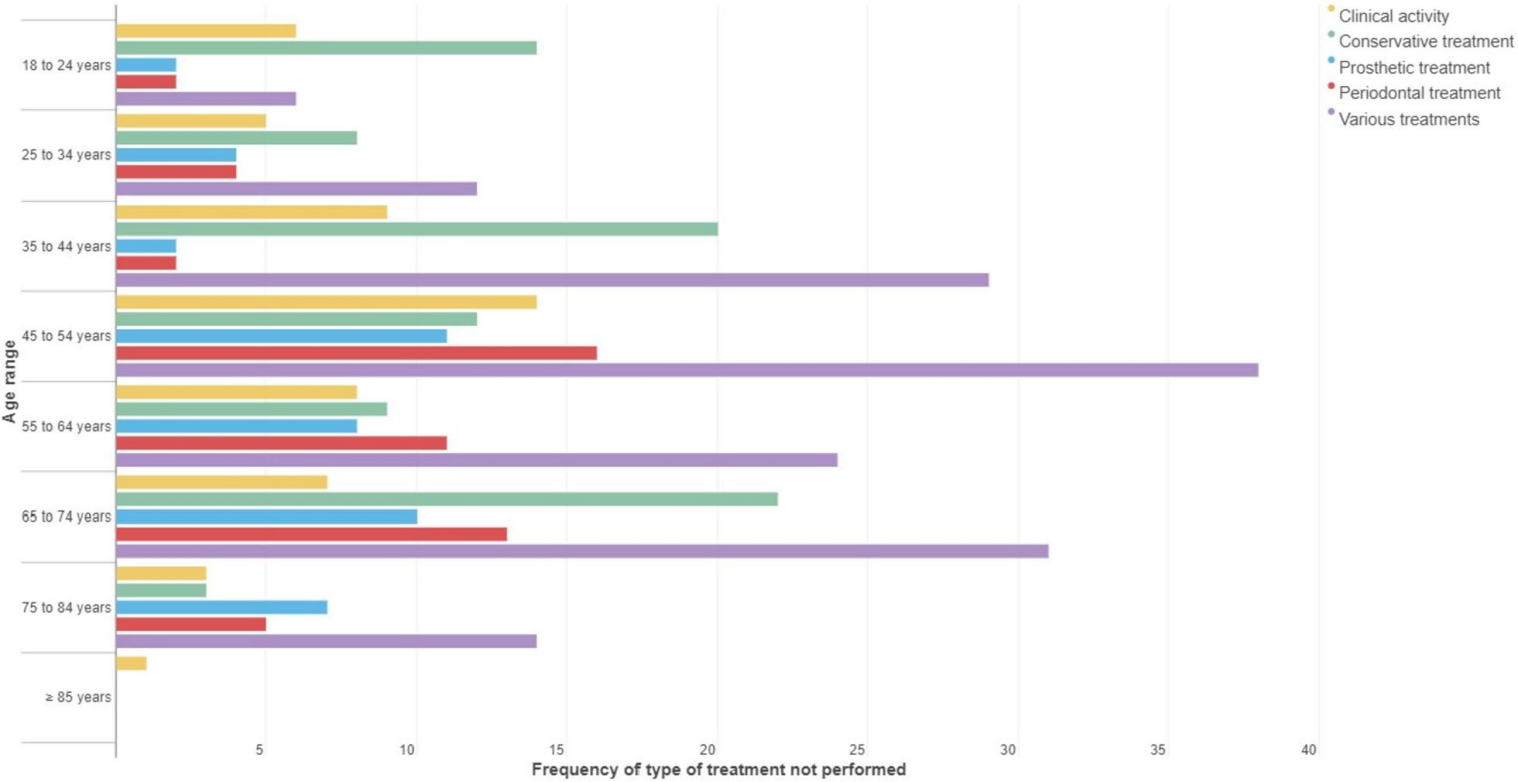
Frequency of the category of treatment not carried out according to the age ranges

Regarding age range, in the subjects between 45 and 64 years and 75 to 84 years, periodontal treatment was the type that most frequently remained unperformed. For the rest of the age ranges, conservative treatment most frequently remained unperformed (Fig. 1).

Of the treatments planned for each participant, in 238 subjects (60.71%), one of the treatment categories was not performed; in 124 subjects (31.63%), two of the treatment categories were not performed and in 30 subjects (7.65%), three of the treatment categories were not performed. The combination of periodontal and conservative treatment recommended for the participants was the type that was not performed most frequently (91 subjects, 23.21%), followed by conservative treatment (88 patients, 22.45%) (Table 3).

**Table 3:** Descriptive statistics of the number of treatment categories not carried out according to the age ranges

Age range (years)	Number of treatment categories not performed															
	CA	%	C	%	Pr	%	Pe	%	C + Pr	%	C + Pe	%	Pe + Pr	%	Pe + Pr + C	%
18–24	6	1.53	14	3.57	2	0.51	2	0.51	1	0.26	5	1.28	0	0.00	0	0.00
25–34	5	1.28	8	2.04	4	1.02	4	1.02	1	0.26	10	2.55	0	0.00	1	0.26
35–44	9	2.30	20	5.10	2	0.51	2	0.51	6	1.53	14	3.57	1	0.26	8	2.04
45–54	14	3.57	12	3.06	11	2.81	16	4.08	6	1.53	22	5.61	3	0.77	7	1.79
55–64	8	2.04	9	2.30	8	2.04	11	2.81	2	0.51	14	3.57	4	1.02	4	1.02
65–74	7	1.79	22	5.61	10	2.55	13	3.32	4	1.02	20	5.10	2	0.51	5	1.28
75–84	3	0.77	3	0.77	7	1.79	5	1.28	1	0.26	6	1.53	2	0.51	5	1.28
≥ 85	1	0.26	0	0.00	0	0.00	0	0.00	0	0.00	0	0.00	0	0.00	0	0.00
Total	53	13.52	88	22.45	44	11.22	53	13.52	21	5.36	91	23.21	12	3.06	30	7.65

C: Conservative treatment; C + Pr: Conservative and prosthetic treatment; C + Pe: Conservative and periodontal treatment; CA: Clinical activities; Pe: Periodontal treatment; Pe + Pr: Periodontal and prosthetic treatment; Pe + Pr + C: Periodontal, prosthetic and conservative treatment; Pr: Prosthetic treatment; %: of the total sample

The multinomial logistic regression model was statistically significant (p = 0.053) and had a low adjustment of the variables (pseudo R^2^ = 5.45%). Only in prosthetic treatment did male sex (OR = 0.42, p = 0.046) act as a protective factor. For the rest of the variables, there was no influence of sex, nationality or age on the number and category of treatments that were not carried out (Table 4).

**Table 4:** Multinomial logistic regression assesses the influence of sex, nationality, and age on the number and categories of treatments that were not performed

Predictor (reference: clinical activities)	Coefficient	SE	OR	*p* value	95% CI
*Conservative treatment*					
Sex (reference: woman)					
Men	− 0.39	0.36	0.68	0.275	(− 1.09 to 0.31)
Nationality (reference: Spanish)					
Foreign	0.03	0.67	1.04	0.958	(− 1.28 to 1.35)
Age range (reference: 18–24)					
25–34	− 0.43	0.75	0.65	0.569	(− 1.91 to 1.05)
35–44	− 0.11	0.64	0.90	0.869	(− 1.35 to 1.14)
45–54	.1.08	0.36	0.34	0.090	(− 2.32 to 0.17)
55–64	− 0.79	0.69	0.46	0.262	(− 2.14 to 0.58)
65–74	0.27	0.66	1.32	0.677	(− 1.02 to 1.57)
75–84	− 0.88	0.96	0.41	0.356	(− 2.76 to 0.99)
≥ 85	− 21.21	28,585.88	0.00	0.999	(− 56,048.49 to 56,006.08)
*Prosthetic treatment*					
Sex (reference: woman)					
Men	− 0.86	0.43	0.42	0.046	(− 1.72− − 0.14)
Nationality (reference: Spanish)					
Foreign	0.31	0.83	1.36	0.710	(− 1.31 to 1.92)
Age range (reference: 18–24)					
25–34	0.75	1.06	2.12	0.479	(− 1.33 to 2.84)
35–44	− 0.53	1.14	0.59	0.644	(− 2.75 to 1.70)
45–54	0.72	0.92	2.05	0.436	(− 1.09 to 2.52)
55–64	1.02	0.97	2.78	0.292	(− 0.88 to 2.92)
65–74	1.44	0.97	4.21	0.137	(− 0.46 to 3.33)
75–84	1.90	1.08	6.68	0.079	(− 0.22 to 4.02)
≥ 85	− 19.67	40,426.53	0.00	1.000	(− 79,254.22 to 79,214.88)
*Periodontal treatment*					
Sex (reference: woman)					
Men	− 0.24	0.40	0.79	0.546	(− 1.02 to 0.54)
Nationality (reference: Spanish)					
Foreign	− 1.10	1.15	0.33	0.339	(− 3.36 to 1.16)
Age range (reference: 18–24)					
25–34	0.85	1.06	2.34	0.421	(− 1.23 to 2.93)
35–44	− 0.45	1.13	0.64	0.693	(− 2.67 to 1.77)
45–54	1.15	0.90	3.14	0.203	(− 0.62 to 2.91)
55–64	1.32	0.94	3.73	0.163	(− 0.53 to 3.17)
65–74	1.62	0.95	5.05	0.087	(− 0.23 to 3.47)
75–84	1.51	1.10	4.53	0.170	(− 0.64 to 3.66)
≥ 85	− 19.93	36,834.51	0.00	1.000	(− 72,214.24 to 72,174.39)
*Conservative and prosthetic treatments*					
Sex (reference: woman)					
Men	0.45	0.53	1.57	0.396	(− 0.59 to 1.50)
Nationality (reference: Spanish)					
Foreign	− 0.52	1.17	0.60	0.657	(− 2.80 to 1.77)
Age range (reference: 18–24)					
25–34	0.24	1.54	1.27	0.878	(− 2.78 to 3.26)
35–44	1.44	1.21	4.24	0.231	(− 0.92 to 3.81)
45–54	1.01	1.19	2.75	0.395	(− 1.32 to 3.35)
55–64	0.43	1.34	1.54	0.749	(− 2.20 to 3.06)
65–74	1.22	1.25	3.38	0.332	(− 1.24 to 3.67)
75–84	0.69	1.59	1.99	0.664	(− 2.42 to 3.80)
≥ 85	− 20.35	58,517.15	0.00	1.000	(− 114,711.9 to 114,671.2)
*Conservative and periodontal treatments*					
Sex (reference: woman)					
Men	− 0.46	0.35	0.63	0.191	(− 1.16 to 0.23)
Nationality (reference: Spanish)					
Foreign	0.31	0.65	1.37	0.632	(− 0.97 to 1.60)
Age range (reference: 18–24)					
25–34	0.81	0.82	2.24	0.325	(− 0.80 to 2.42)
35–44	0.56	0.75	1.75	0.452	(− 0.90 to 2.02)
45–54	0.57	0.70	1.76	0.420	(− 0.81 to 1.94)
55–64	0.72	0.76	2.05	0.344	(− 0.77 to 2.20)
65–74	1.24	0.76	3.46	0.100	(− 0.24 to 2.72)
75–84	0.87	0.94	2.38	0.354	(− 0.97 to 2.71)
≥ 85	− 20.07	28,110.73	0.00	0.999	(− 55,116.09 to 55,075.95)
*Periodontal and prosthetic treatments*					
Sex (reference: woman)					
Men	− 1.30	0.75	0.27	0.082	(− 2.77 to 0.16)
Nationality (reference: Spanish)					
Foreign	1.79	1.03	5.60	0.081	(− 0.22 to 3.81)
Age range (reference: 18–24)					
25–34	0.28	1976.04	1.32	1.000	(− 3872.69 to 3873.24)
35–44	14.10	1514.02	1,330,626.00	0.993	(− 2953.32 to 2981.52)
45–54	14.10	1514.02	3,037,930.00	0.992	(− 2952.49 to 2982.34)
55–64	16.02	1514.02	9,078,710.00	0.992	(− 2951.39 to 2983.44)
65–74	15.58	1514.02	5,853,548.00	0.992	(− 2951.83 to 2982.10)
75–84	16.29	1514.02	0.00	0.991	(− 2951.13 to 2983.71)
≥ 85	− 4.89	77,425.72	0.01	1.000	(− 151,756.5 to 15,146.7)
*Periodontal, prosthetic and conservative treatments*					
Sex (reference: woman)					
Men	− 0.18	0.47	0.84	0.700	(− 1.09 to 0.74)
Nationality (reference: Spanish)					
Foreign	0.06	0.93	1.06	0.949	(− 1.75 to 1.87)
Age range (reference: 18–24)					
25–34	14.25	1150.63	1,550,899.00	0.990	(− 2240.94 to 2269.45)
35–44	15.74	1150.63	6,883,666.00	0.989	(− 2239.45 to 2270.94)
45–54	15.16	1150.63	3,848,117.00	0.989	(− 2240.03 to 2270.36)
55–64	15.18	1150.63	3,899,838.00	0.989	(− 2240.02 to 2270.37)
65–74	15.55	1150.63	5,642,544.00	0.989	(− 2239.65 to 2270.74)
75–84	16.39	1150.63	0.00	0.989	(− 2238.81 to 2271.58)
≥ 85	− 5.63	48,972.48	0.00	1.000	(− 95,989.93 to 95,978.67)

Data of the model: n = 392, pseudo R^2^ = 5.45%, *p* value = 0.053. SE: Standard error; CI: Confidence interval; OR: Odds ratio

## Discussion

The general findings of this study show that conservative treatments were the most affected procedures during the COVID-19 pandemic, regardless of the age interval, followed by periodontal treatment, where almost half of the planned treatments could not be performed. However, 86.48% of the patients in the sample could be treated in other clinical activities, including dental assessments, check-ups, and emergencies.

Since routine care in dental clinics was suspended during the first wave of the pandemic, the undiagnosed or uncontrolled progression of the dental diseases described could be anticipated [19]. The foresight in the reassessment of a patient before the cessation of conservative treatments, such as fillings or endodontics, and the case of neglect of periodontal treatments, such as periodontal study, scaling and root planing, periodontal reassessment, periodontal maintenance, extractions, gingivectomies or coronary lengthening, would fundamentally lead to a progression of periodontal disease. It should be taken into account that the most frequent reasons for tooth extraction in teeth with untreated disease are caries, dental fractures, and periodontal disease [22]. The recent systematic review by Broers et al*.* [23] shows that the percentage of dental extractions (more radical treatment) due to dental caries is 36% to 55.3%, and for periodontitis, from 24.8% to 38.1%. For this reason, follow-up in the dental planning of the patients led to the negative effects described.

On the other hand, the age groups most affected by the cessation of activity and the absence of conservative and periodontal treatments were patients aged between 35 and 74 years.

Mobility restrictions and the confinement measures were generally applied standards throughout the population [23, 24]. Therefore, it is logical in our study to find that there is no influence of age, sex, or nationality on the number and type of dental treatments that were not performed in patients.

The negative impact of the COVID-19 pandemic has been evaluated in the pediatric dentistry services of the Brazilian Public Health System [25], which services patients with special needs [26], and its effects on teaching at the educational level. Although academic performance has not been quantified in our study, the number of patients seen by fifth-graders was lower than in other academic years, which may have had a negative impact. Dentistry education was regulated under the management of patients [27, 28], but none of them discriminated by the types of treatments that were not performed. The strengths of this study lie in showing the frequency of planned and unperformed treatments.

Additionally, it must be taken into account that although the number of registered patients in all the services of the FU-URJC (such as the Dental Clinic of Special Patients and Gerontology and Integrated Children's Dental Clinic of the Degree of Dentistry), was 56% lower in the year the pandemic started compared to the previous academic year (the academic year 2018–2019: 14,949 citations, compared to the academic year 2019–2020: 8,372 citations). Although the number of patients analyzed was less than that of the previous academic year, the sample turned out to be sufficiently representative.

It should also be taken into account that this restrospective study only reports on the number of treatments not performed since it was not possible to collect in all cases the information on the reasons why the patient could not attend the appointment. However, the trend and negative impact on dental care is observed during the onset of the pandemic.

## Conclusions

The COVID-19 pandemic could have negatively influenced treatments, such as conservative and periodontal treatments, that increasing the risk of tooth loss in adults.

## Data Availability

Data are available from the corresponding author on reasonable request.

## Abbreviations

BOE: Spain's Official State Gazette
COVID-19: Coronavirus Disease 2019
FU-URJC: Fundación Clínica Universitaria of the Rey Juan Carlos University
SCBS: The Ministry of Health, Consumption and Social Welfare
Spanish NHS: Spanish National Health Survey
STROBE: Strengthening the Reporting of Observational Studies in Epidemiology
URJC: Rey Juan Carlos University
WHO: World Health Organization
